# Preoperative serum level of CA153 and a new model to predict the sub-optimal primary debulking surgery in patients with advanced epithelial ovarian cancer

**DOI:** 10.1186/s12957-024-03336-2

**Published:** 2024-02-23

**Authors:** Yue Jia, Yaping Jiang, Xiaoqi Fan, Ya Zhang, Kun Li, Haohan Wang, Xianling Ning, Xielan Yang

**Affiliations:** 1 Department of Gynecology, The Third Affiliated Hospital of Kunming Medical University, Yunnan Cancer Hospital, Kunming, Yunnan P. R. China 650118; 2 Department of Radiology, The Third Affiliated Hospital of Kunming Medical University, Yunnan Cancer Hospital, Yunnan Cancer Center, Kunming, Yunnan P. R. China 650118

**Keywords:** Advanced epithelial ovarian cancer, Prediction model, Primary debulking surgery, Computed tomography, CA153

## Abstract

**Objective:**

The aim of this study was to establish a preoperative model to predict the outcome of primary debulking surgery (PDS) for advanced ovarian cancer (AOC) patients by combing Suidan predictive model with HE4, CA125, CA153 and ROMA index.

**Methods:**

76 AOC Patients in revised 2014 International Federation of Gynecology and Obstetrics (FIGO) stage III-IV who underwent PDS between 2017 and 2019 from Yunnan Cancer Hospital were included. Clinical data including the levels of preoperative serum HE4, CA125, CA153 and mid-lower abdominal CT-enhanced scan results were collected. The logistics regression analysis was performed to find factors associated with sub-optimal debulking surgery (SDS). The receiver operating characteristic curve was used to evaluate the predictive performances of selected variables in the outcome of primary debulking surgery. The predictive index value (PIV) model was constructed to predict the outcome of SDS.

**Results:**

Optimal surgical cytoreduction was achieved in 61.84% (47/76) patients. The value for CA125, HE4, CA153, ROMA index and Suidan score was lower in optimal debulking surgery (ODS) group than SDS group. Based on the Youden index, which is widely used for evaluating the performance of predictive models, the best cutoff point for the preoperative serum HE4, CA125, CA153, ROMA index and Suidan score to distinguish SDS were 431.55 pmol/l, 2277 KU/L, 57.19 KU/L, 97.525% and 2.5, respectively. Patients with PIV≥5 may not be able to achieve optimal surgical cytoreduction. The diagnostic accuracy, NPV, PPV and specificity for diagnosing SDS were 73.7%, 82.9%, 62.9% and 72.3%, respectively. In the constructed model, the AUC of the SDS prediction was 0.770 (95% confidence interval: 0.654-0.887), *P*<0.001.

**Conclusion:**

Preoperative serum CA153 level is an important non-invasive predictor of primary SDS in advanced AOC, which has not been reported before. The constructed PIV model based on Suidan's predictive model plus HE4, CA125, CA153 and ROMA index can noninvasively predict SDS in AOC patients, the accuracy of this prediction model still needs to be validated in future studies.

## Introduction

Ovarian cancer is the most lethal malignant carcinoma of female reproductive system, with an estimated 27,200 deaths in 2016 in China [1] and 207,252 deaths in 2020 worldwide [2]. More than 75% of ovarian cancer patients are initially diagnosed as an advanced stage due to insidious onset and less obvious symptoms at the early stage. Currently, cytoreductive surgery and platinum-based chemotherapy are the priority options recommended by guidelines for the treatment of ovarian cancer. Optimal cytoreductive surgery is the well-known means to bring the best prognosis for the patients. Therefore, the precise prediction outcome of cytoreductive surgery is clinically useful and meaningful.

Previous studies [3, 4] have proved that the size of residual disease (RD) after surgery is the most important independent prognostic factor for AOC patients, and the residual lesions less than 1 cm can significantly improve the prognosis of patients [5, 6]. Therefore, the maximum diameter of RD less than 1 cm was defined as optimal debulking surgery (ODS) [7]. However, it is difficult to achieve optimal cytoreduction for some AOC patients due to the lesions widely spread in the pelvic and abdominal cavity and the tumor closely adhere to surrounding tissues when diagnosed. Some scholars [8–10] have found that neoadjuvant chemotherapy (NACT) is beneficial to achieve optimal cytoreduction. However, other scholars [9, 11–13] have suggested that NACT instead of PDS has no benefit in over-all survival for AOC patients, which indicated the importance of PDS in patients with AOC. As the existence of controversial views, it is difficult for gynecologic oncologists to select an appropriate initial treatment between NACT and PDS. Therefore, the prediction of PDS outcome is not only conducive to more adequate preoperative preparation, but also provide basis for patients to choose PDS or NACT [14, 15].

From the perspective that AOC patients can benefit from ODS, it is important to predict AOC patients who will not be able to achieve optimal cytoreduction. In fact, many predictive models have been established to evaluate the outcome of PDS, such as tumor biomarker [16], frailty index [17], diagnostic imaging [18] and laparoscopic findings [19, 20]. In some studies [16, 21, 22], Carcinoembryonic Antigen 125 (CA125) and human epididymis protein 4 (HE4) have been used to predict the sub-optimal debulking surgery (SDS). In other studies [18, 23], computed tomography (CT) scan findings or CT-based predictive model were also used for the prediction of SDS. In addition, risk of ovarian malignancy algorithm (ROMA) value plus CA125, HE4 and menopausal status was combined to establish a prediction model of the sub-optimal debulking surgery [24]. Although many efforts have been made to improve the predictive efficacy of SDS, there is still a need to develop more effective methods.

In the present study, we retrospectively analyzed the predictive value of screened tumor biomarker (CA125, HE4 and CA153), Suidan score and ROMA index alone or in combination in predicting the outcome of PDS. The purpose of this study was to develop a reliable noninvasive scoring system for predicting the SDS in patients with AOC.

## Patients and Methods

### Patients

**Fig. 1:**
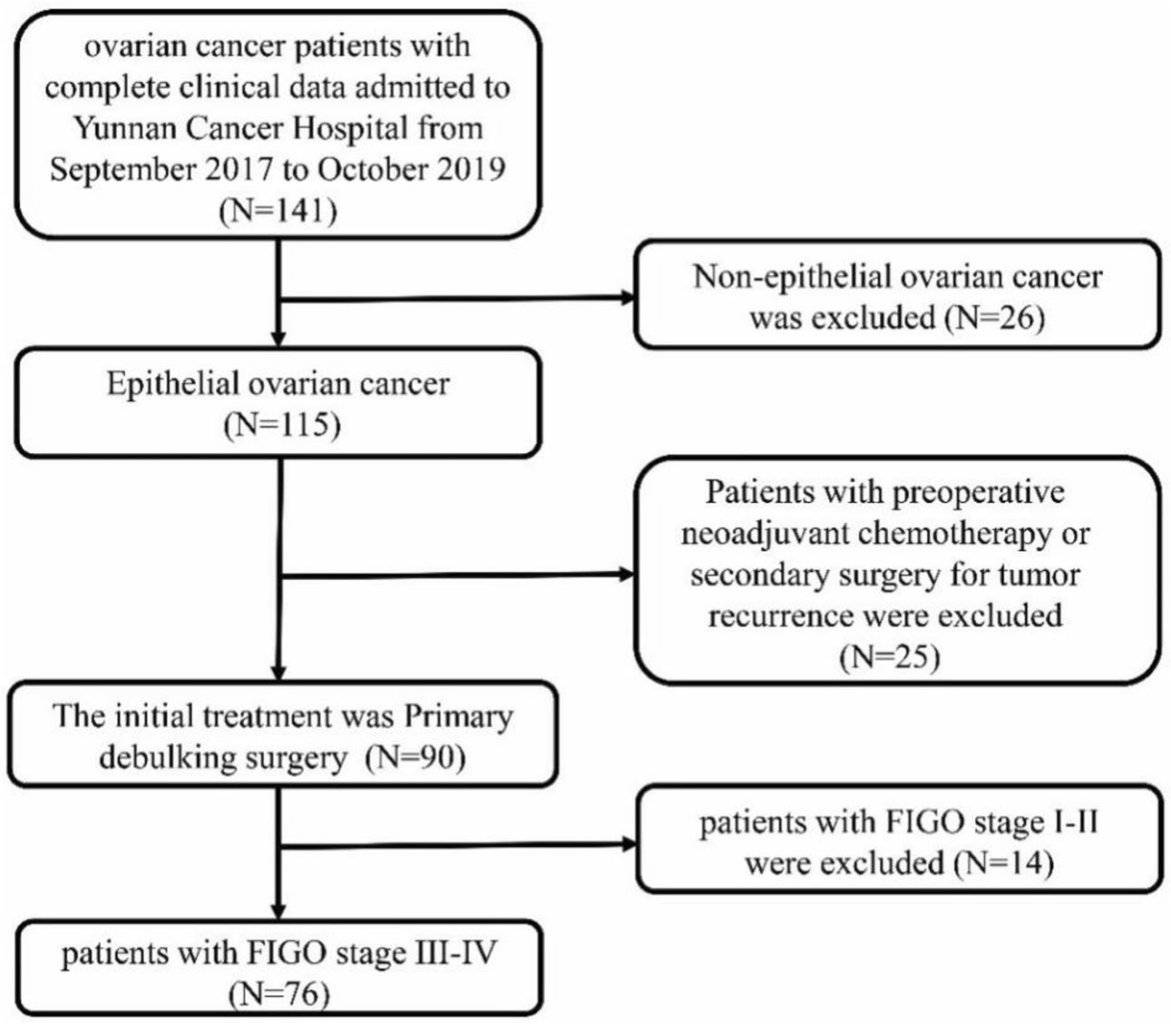
Flow chart of patient selection

A retrospective analysis of ovarian cancer patients in the Yunnan cancer hospital from September 2017 to October 2019 was performed. The inclusion and exclusion criteria are as following: Inclusion criteria: 1) patients with complete clinical data including age, preoperative serum CA125, CA153 and HE4 level, ROMA, preoperative mid-lower abdominal CT-enhanced scan results, histological type, FIGO stage, grade, and surgical results etc; 2) patients who underwent PDS at Yunnan cancer hospital; 3) patients with pathologically confirmed epithelial ovarian carcinoma; 4) patients with FIGO stage III or IV. Exclusion criteria: 1) patients who received targeted therapy and NACT before PDS; 2) patients with secondary tumor recurrence. The patient selection process is shown in Fig. 1. According to inclusion and exclusion criteria, 76 patients were enrolled in the study. This study was approved by the Committee at Yunnan cancer hospital, and the approved number is KYLX202174. The informed consent was obtained from all enrolled subjects.

### Definition of ODS and SDS

The outcome of PDS was determined as follows: no gross RD or RD less than 1 cm in maximum tumor diameter was defined as ODS, and RD more than 1 cm in maximum tumor diameter was defined as SDS.

### Data Collection

The demographics of patients and the value of preoperative serum level were obtained from medical records management system. The ROMA was determined based on the patients’ menstrual status and the value of preoperative serum HE4 and CA125 according to previous literature calculation methods [24]. The Suidan score was also determined based on previous literature [25], including 3 clinical factors and 8 imaging indexes of CT. The image analysis of pelvic and abdominal cavity enhanced CT scanning was determined by two independent imaging experts in our hospital. The data of this study is available from the corresponding author upon reasonable request.

### Statistical analysis

Statistical analysis was performed using IBM SPSS 26.0 and MedCalc 18.2.1. The categorical variables were described as frequencies with percentages. Continuous variables were first tested for normal distribution, non-normally distributed continuous variables were represented as medians with ranges. The Mann-Whitney U test was used to compare statistical differences of selected variables between ODS and SDS group. The receiver operating characteristic curve was applied to evaluate the performances of selected variables in predicting the outcome of PDS. The area under the curve (AUC) values were evaluated as an indicator for predictive accuracy. The cutoff value of each selected variable was determined with the corresponding ROC curve. The sensitivity, specificity, positive predictive value (PPV), negative predictive value (NPV) and accuracy of each selected variable and prediction model were calculated according to different cutoff values. Logistic regression analysis was used to find factors associated with SDS. The predictive value between prediction model and selected variable was determined using MedCalc 18.2.1 software, and P<0.05 (two-sided) was considered to be statistically different.

## Results

### Clinical data of the patients with advanced ovarian cancer

**Fig. 2:**
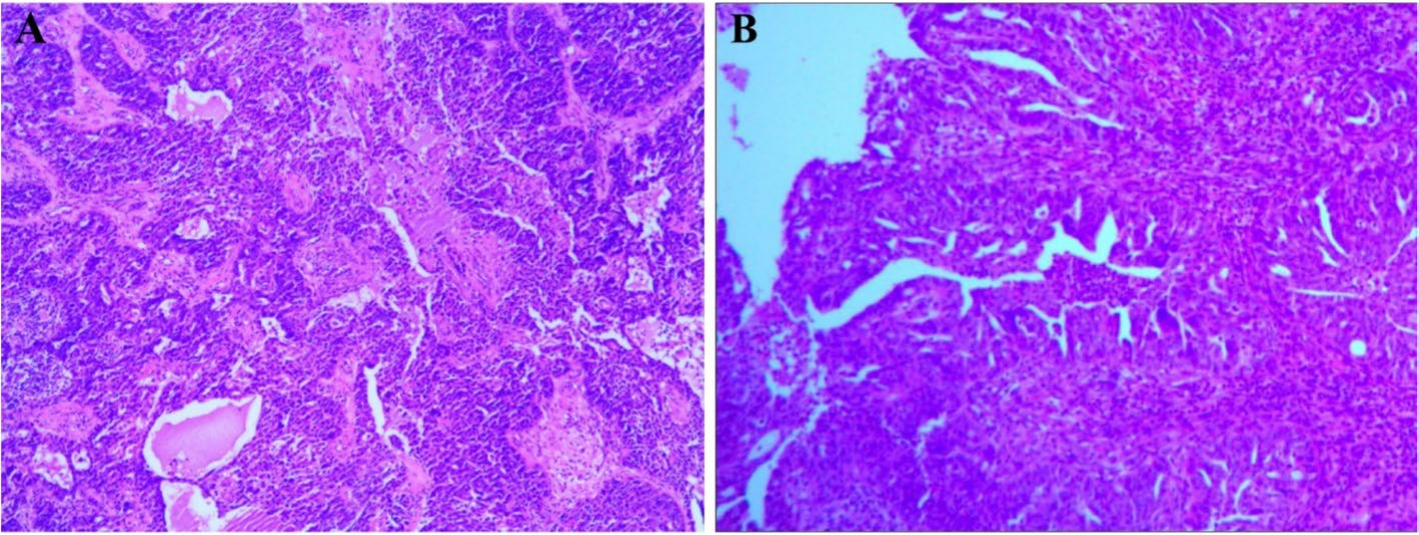
Representative histological images **A** H&E staining (Original magnification: 40×) for ovary tissue; **B** H&E staining (Original magnification: 40×) for Metastatic foci;

A total of 76 patients who met the inclusion criteria were enrolled from September 2017 to October 2019. The clinical characteristics of patients were summarized in Table 1. The age of patients less than 50 years and greater than or equal to 50 years accounted for 32.9% and 67.1% respectively. Surprisingly, the histological type of all patients in this study was serous adenocarcinoma (Fig. 2). According to FIGO staging, there were 58 patients in stage III and 18 patients in stage IV. In all patients, the histological grade of 72 patients were at high grade, another 2 patients were at low grade, and 2 patients were unknown. The primary ovarian cancer site at ovary and the fallopian tubes accounted for 96.1% and 3.9% respectively. After surgery, 47 patients were achieved ODS and 29 patients did not achieve ODS.

**Table 1:** Patients Characteristics (N=76)

Characteristics	*N*	%
Age		
<50	25	32.9
≥50	51	67.1
The primary site		
ovary	73	96.1
The fallopian tubes	3	3.9
Residual disease		
<1 cm	47	61.8
≥1 cm	29	38.2
FIGO stage		
III A/B	16	21.1
III C	42	55.2
IV	18	23.7
Histological type		
Serous adenocarcinoma	76	100
Histological grade		
grade 1	2	2.6
grade 2	1	1.3
grade 3	71	93.5
Unknown	2	2.6

### The different variables were compared according to surgical outcome

Related clinical preoperative factors were analyzed between ODS and SDS group in this study. Table 2 showed the statistically different variables between ODS and SDS group. The level of serum HE4, CA125 and CA153 were all significantly elevated in SDS group compared to ODS group (*P<*0.05). The ROMA index and the median of Suidan score were both increased in SDS group compared to ODS group (*P<*0.01). These data suggested that these variables might play an important role in predicting surgical outcome.

**Table 2:** The preoperative HE4, CA125 and CA153, Suidan score and ROMA index between ODS and SDS group (N=76)

Variables	ODS group (N=47)	SDS group (N=29)	z	*p*
HE4 (pmol/l)	407.3 (189.9, 626.2)	661.1 (448.1, 1186.0)	-2.818	0.005
CA125 (KU/L)	1003.3 (386.2, 1703.0)	1443.0 (534.7, 2744.5)	-1.877	0.061
CA153 (KU/L)	51.6 (30.5, 143.2)	100.2 (51.4, 202.9)	-2.101	0.036
Suidan score	2 (1, 3)	3, (2, 5.5)	-2.746	0.006
Roma index (%)	95.82 (76.51, 97.72)	97.96 (93.45, 99.46)	-2.914	0.004

### Diagnostic efficacy of selected variables in predicting SDS

To further analyze the predictive value of HE4, CA125, CA153, ROMA index and Suidan score for SDS in AOC patients, ROC curve was applied to analyze the above indicators, and the maximum value of Youden index was taken as the cutoff value. Table 3 showed the sensitivity, specificity, PPV, NPV and accuracy of each variable in predicting SDS. The ROC analysis results indicated that HE4, CA153, ROMA index and Suidan score were significantly correlated with SDS. The AUC for HE4, CA125, CA153, ROMA index and Suidan score were 0.693 (P=0.005), 0.629 (P=0.061), 0.644 (P=0.036), 0.7 (P=0.007) and 0.685 (P=0.004) respectively. And the value of cutoff for HE4, CA125, CA153, ROMA index and Suidan score were 431.55 pmol/l, 2277 KU/L, 57.19 KU/L, 97.525% and 2.5 respectively.

**Table 3:** Predictive efficacy of selected variables for sub-optimal debulking surgery

Variables	Sensitivity	Specificity	PPV	NPV	Accuracy	AUC (95% CI)	*p*	Cutoff
HE4 (pmol/l)	79.3%	53.2%	51.1%	80.6%	63.2%	0.693 (0.570, 0.817)	0.005	431.6
CA125 (KU/L)	37.9%	87.2%	64.7%	69.5%	68.4%	0.629 (0.498, 0.760)	0.061	2277.0
CA153 (KU/L)	75.9%	57.4%	52.4%	79.4%	64.5%	0.644 (0.513, 0.775)	0.036	57.2
Suidan score	58.6%	74.5%	58.6%	74.5%	68.4%	0.685 (0.561, 0.810)	0.007	2.5
ROMA index	62.1%	72.3%	58.1%	75.6%	68.4%	0.700 (0.577, 0.823)	0.004	97.5%
Prediction model	0.759	0.723	0.629	0.829	0.737	0.770 (0.654, 0.887)	<0.001	5

### Logistic regression for evaluation of SDS prediction model

Table 4 showed the univariate logistic regression analysis of selected variables for predicting SDS outcomes. The results demonstrated that the variables selected were all associated with SDS. HE4 level>431.55 pmol/l (OR=4.356, 95% CI 1.501-12.644, P=0.007), CA125 level>2277 KU/L (OR=4.176, 95% CI 1.337-13.040, P=0.014), CA153 level>57.19 KU/L (OR=2.06, 95% CI 1.232-3.445, P=0.004), ROMA index>97.525% (OR=2.069, 95% CI 1.264-3.386, P=0.004) and Suidan scoring>2 (OR=2.033, 95% CI 1.240-3.331, P=0.005) were all significantly increased the risk of SDS.

**Table 4:** The univariate analysis of selected variables in predicting sub-optimal debulking surgery

Variables	N	B	OR	95%CI	*p*
Preoperative HE4		1.472			0.007
≤431.55 pmol/l	31		1		
>431.55 pmol/l	45		4.356	1.501-12.644	
Preoperative CA125		1.429			0.014
≤2277 KU/L	59		1		
>2277 KU/L	17		4.176	1.337-13.040	
Preoperative CA153		0.723			0.006
≤57.19 KU/L	34		1		
>57.19 KU/L	42		2.06	1.232-3.445	
Rome index		0.727			0.004
≤97.525	45		1		
>97.525	31		2.069	1.264-3.386	
Suidan Scoring		0.709			0.005
≤2	47		1		
>2	29		2.033	1.240-3.331	

### Predictive index value (PIV) model for predicting SDS

**Fig. 3:**
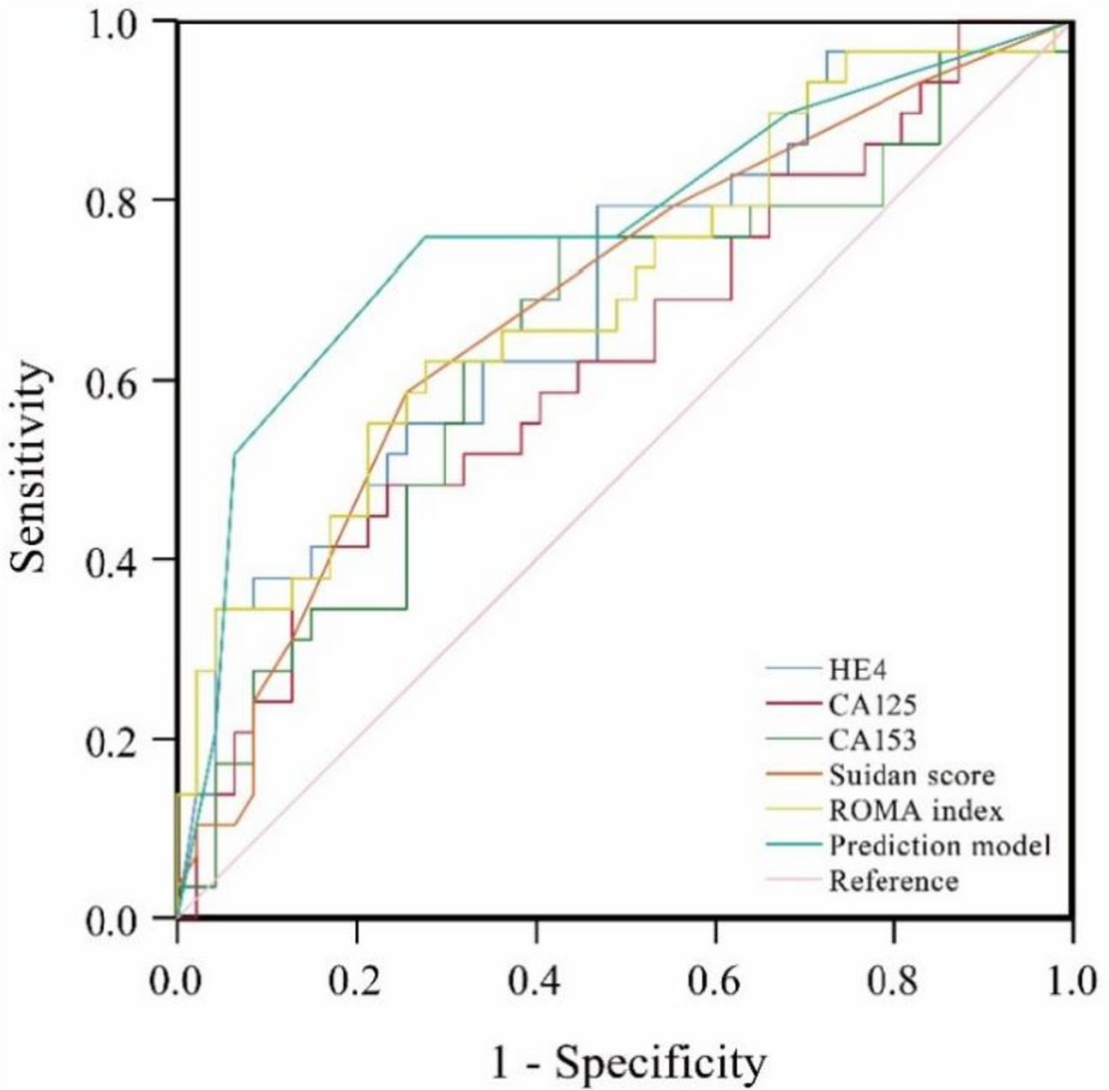
ROC curve of predicted probability of selected variables and prediction model for sub-optimal debulking surgery

Parameters meeting accuracy≥75%, PPV≥50% and NPV≥50% were included in the PIV model, and each parameter was scored 1 point. The PIV ranged from 0 to 10 points. Table 5 showed the PIV prediction model under different cutoffs. The sensitivity, specificity, PPV, NPV and accuracy of each PIV were determined. The PIV model showed that the diagnostic sensitivity became lower and the specificity became higher as the PIV score increased (Table 5). Therefore, to maximize prediction accuracy and minimize the incidence of inappropriate exploration, PIVs greater than 7 achieved the highest accuracy of 77.6% and identified patients receiving SDS with 93.6% specificity. The ROC curve of the PIV prediction model was shown as Fig. 3. The AUC of PIV for predicting SDS was 0.770 (Table 5, greater than 0.75), and the cutoff value was 5, suggesting that this prediction model had discriminative power, and SDS was more likely to achieve when PIV greater than 5. The Hosmer-Lemeshow test (χ^2^=9.458, P>0.05) indicated that the variables were well fit the logistic regression model. In order to evaluate whether the PIV prediction model had a better prediction value than single selected variable, we compared the AUC curve between the prediction model and the single variable. The results displayed that the prediction model has a significantly prediction value than CA153. Although the AUC of prediction model was higher than HE4 and CA125, there was not a statistically difference.

**Table 5:** The overall prediction model according to different cutoff values

PIV	Sensitivity	Specificity	PPV	NPV	Accuracy
≥0	100.0%	0	38.2%	N/A	38.2%
≥1	89.7%	31.9%	44.8%	83.3%	53.9%
≥3	75.9%	51.1%	48.9%	77.4%	60.5%
≥5	75.9%	72.3%	62.9%	82.9%	73.7%
≥7	51.7%	93.6%	83.3%	75.9%	77.6%
≥9	20.7%	95.7%	75.0%	66.2%	67.1%

## Discussion

In various studies [26–28], it has been reported that AOC patients can benefit from optimal debulking surgery. In this study, we sought to develop a reliable noninvasive scoring system for predicting sub-optimal debulking surgery in patients with AOC. We found that our predictive model was helpful in predicting SDS in AOC patients, with predictive sensitivity, specificity and accuracy of 0.759, 0.723 and 0.737 respectively. The AUC of the constructed model for the SDS was 0.770 (95% confidence interval: 0.654-0.887), P<0.001. Furthermore, the value for selected tumor biomarker (CA125, HE4 and CA153), ROMA index and Suidan score was lower in ODS group than SDS group.

According to our collected data, we found that the tumor biomarkers of HE4 and CA153 had the significant predictive value for sub-optimal debulking surgery in AOC patients. CA125 and HE4 had been reported in predicting sub-optimal debulking surgery in previous studies [29–31]. However, the ROC analysis showed that CA125 could not predict sub-optimal debulking surgery in this study. CA125 was used for auxiliary diagnosis and postoperative recurrence monitoring of ovarian cancer [32]. In most studies [25, 29, 33], the cutoff of CA125 for predicting SDS was about 500 U/ml, while, the cutoff value in this study astonishingly reached 2277 U/ml, which may be correlated with the pathological types and stages of enrolled patients. In contrast, univariate logistic regression analysis showed that CA125 greater than 2277 U/ml was associated with sub-optimal cytoreduction (P=0.014). Considering the importance of CA125 in predicting sub-optimal cytoreduction, we also selected it in our constructed model.

HE4 had been routinely determined as a tumor marker in diagnosis, prognosis assessment, recurrence, chemotherapy sensitivity and follow-up after the treatment of ovarian cancer [34]. Several studies [34–36] had reported that HE4 could predict the surgical outcome after PDS in AOC patients, which was in consistent with our study. Tang et al. [36] showed that the optimal cutoff value for HE4 to predict SDS of AOC patients was 473 pmol/L. Our results showed that the optimal cutoff value for HE4 to predict SDS of AOC patients was 431.55 pmol/L, which was consistent with previous study. When the cutoff value of HE4 was higher than 431.55 pmol/L, the sensitivity, specificity, and AUC for predicting SDS were 0.80, 0.53 and 0.693 respectively. Unexpectedly, we also found the predictive value of CA153 for sub-optimal debulking surgery, which has never been reported in the previous studies. The univariate logistic regression analysis showed that CA153 could predict SDS in AOC patients (P=0.006), and the AUC of CA153 in predicting SDS was 0.644, which was even higher than the AUC of CA125. However, it still needed to be confirmed whether CA153 can be widely used as an effective predictor in predicting SDS.

The ROMA index was calculated for each patient based on CA125, HE4 and the patient's menopausal status. The univariate logistics regression analysis approved that ROMA index was a valuable predictor in SDS in AOC patients (P=0.005), and the AUC of ROMA index in predicting SDS was 0.700. These results indicated that ROMA may play a more important role in predicting the surgical outcome of AOC patients. CT had significant advantages in assessing the size, morphology, surrounding invasion and distant metastasis of ovarian cancer, and has been widely used in preoperative examination of ovarian cancer [37]. Several studies [38–40] on the CT-based prediction of the surgical outcome had been reported. The constructed model for predicting the surgical outcome by Suidan et al. [25] was considered as the most quantitative prediction model to date. In our study, we also verified the predictive value of Suidan model. In the ROC analysis, the AUC of predicting SDS by Suidan model was 0.685, and the cutoff value was 2, which was in consistent was previous studies.

In the present study, the preoperative HE4, CA125, CA153 and ROMA index were incorporated into Suidan model to verify whether they could improve the predictive power of SDS. Our scoring model showed that with the increase of PIV score, the sensitivity decreased while the specificity increased. When the PIV score was equal or greater than 7, the specificity and the accuracy for predicting SDS was 93.6% and 77.6%. In some cases, if the SDS was predicted, 3 to 6 courses of neoadjuvant chemotherapy were considered to perform before internal debulking surgery [41]. The histological type of the primary tumor is considered to be the most important factor determining the suitability of NACT. It is best to obtain histological evidence through puncture biopsy or laparoscopic surgery before NACT. However, the positive of ascites cytological combined with the ratio of CA125 to CEA greater than 25 can replace puncture biopsy or laparoscopic surgery when it is difficult to obtain histological evidence [41, 42]. In other cases, if the surgical outcome of primary debulking surgery was difficult to determine, Fagotti's PIV model can also be considered [43]. Although the model we constructed exhibited a good predictive value, there are still some deficiencies in the study design. For example, this is a retrospective study with a small sample size, and there are some subjective differences in CT scores, etc. Moreover, we did not conduct internal verification of the constructed model, so the predictive efficacy of the constructed model still needed to be further verified.

## Conclusion

Preoperative serum CA153 level is an important non-invasive predictor of primary SDS in advanced AOC, which has not been reported before. We constructed a noninvasively sub-optimal debulking surgery prediction model in AOC patients based on Suidan's predictive model plus HE4, CA125, CA153 and ROMA index, which provides an available and meaningful tool for identifying patients who are not eligible for primary debulking surgery.

## Data Availability

The datasets analyzed in this study are not publicly available. However, they can be available from the corresponding author on reasonable requests.
